# Large abdominal mechanoreceptive sense organs in small plant-dwelling insects

**DOI:** 10.1098/rsbl.2022.0078

**Published:** 2022-04-13

**Authors:** Sarah Ehlers, Daniel Baum, Roland Mühlethaler, Hannelore Hoch, Peter Bräunig

**Affiliations:** ^1^ Centre for Integrative Biodiversity Discovery (CIBD), Museum of Natural History Berlin (MfN), Invalidenstraße 43, 10115 Berlin, Germany; ^2^ Visual and Data-Centric Computing, Zuse Institute Berlin, Takustr. 7, 14195 Berlin, Germany; ^3^ Wunsiedeler Weg 36, 12247 Berlin, Germany; ^4^ Centre for Integrative Biodiversity Discovery (CIBD), Museum of Natural History Berlin, Invalidenstraße 43, 10115 Berlin, Germany; ^5^ Biology Department II (Zoology), RWTH Aachen University, Worringerweg 3, 52074 Aachen, Germany

**Keywords:** biotremology, hemiptera, insect communication, chordotonal organs, sensory evolution, vibration detection

## Abstract

The Hemiptera, with approximately 98 000 species, is one of the largest insect orders. Most species feed by sucking sap from plant tissues and are thus often vectors for economically important phytopathogens. Well known within this group are the large cicadas (Cicadomorpha: Cicadoidea: Cicadidae) because they produce extremely loud airborne sounds. Less well known are their mostly tiny relatives, the leafhoppers, spittlebugs, treehoppers and planthoppers that communicate by silent vibrational signals. While the generation of these signals has been extensively investigated, the mechanisms of their perception are poorly understood. This study provides a complete description and three-dimensional reconstruction of a large and complex array of mechanoreceptors in the first abdominal segments of the Rhododendron leafhopper *Graphocephala fennahi* (Cicadomorpha: Membracoidea: Cicadellidae). Further, we identify homologous organs in the spittlebug *Philaenus spumarius* (Cicadomorpha: Cercopoidea: Aphrophoridae) and the planthopper *Issus coleoptratus* (Fulgoromorpha: Fulgoroidea: Issidae). Such large abdominal sensory arrays have not been found in any other insect orders studied so far. This indicates that these sense organs, together with the signal-producing tymbal organ, constitute a synapomorphy of the Tymbalia (Hemiptera excl. Sternorrhyncha). Our results contribute to the understanding of the evolution from substrate-borne to airborne communication in insects.

## 1. Introduction

In insects, intraspecific communication through substrate-borne vibrational signals is thought to be a phylogenetically ancient trait, dating back at least 300 million years, to the Lower Permian [1,2]. Substrate-borne vibrations are ubiquitous and best studied within the order Hemiptera that comprises approximately 98 000 recent species [3]. Except for the suborder Sternorrhyncha, all other taxa of Hemiptera possess a tymbal organ to generate mechanical signals (sound and/or vibrations) and so have been subsumed under the term ‘Tymbalia’ [4].

Except for cicadas, which communicate by airborne signals, all remaining taxa of Tymbalia communicate by means of substrate-borne vibrations transmitted via the plant surface [5]. These signals are mandatory for the identification and localization of potential mating partners [6]. All species of the suborders Cicadomorpha (leafhoppers, spittlebugs, treehoppers and cicadas) and Fulgoromorpha (planthoppers), belonging to the Tymbalia, use their piercing-sucking mouthparts to feed on sap from plant tissues. For this reason, some of them are very effective vectors for plant pathogens such as phytoplasmas, bacteria and viruses [7,8]. In many cases, these vector-borne plant pathogens are causing massive damage to a broad spectrum of crops, resulting in enormous economic losses [9]. The generation of vibrational signals in Tymbalia is increasingly being studied, especially concerning their production, their role in reproductive behaviour, their ecological contexts and even their application in pest control [10]. By contrast, little is known about how these signals are perceived [11]. Cicadas possess two sound-perceiving abdominal tympanal organs, each containing more than 2000 sensory cells [12]. For leafhoppers, a simple Johnston's organ with only 20 sensory cells or even smaller subgenual organs have been hypothesized to act as the main signal receiver [13,14].

In a publication that went unnoticed for decades, Karel Vondráček [15] reported a signal-producing organ (tymbal) in the leafhopper *Ribautiana ulmi* (Cicadomorpha: Membracoidea: Cicadellidae), and—in addition—observed two paired chordotonal organs (stretch receptor organs with ciliated scolopidial sensory units) in the abdomen that he interpreted as auditory organs. More than 70 years later, our study is the first to follow up and elaborate on Vondráček's observations. Our aim was to bring this pioneering discovery back to light, to analyse the sense organs' structure in detail and provide a basis for comparative studies across a wider range of species within the Tymbalia.

## 2. Material and methods

**Figure 1.**
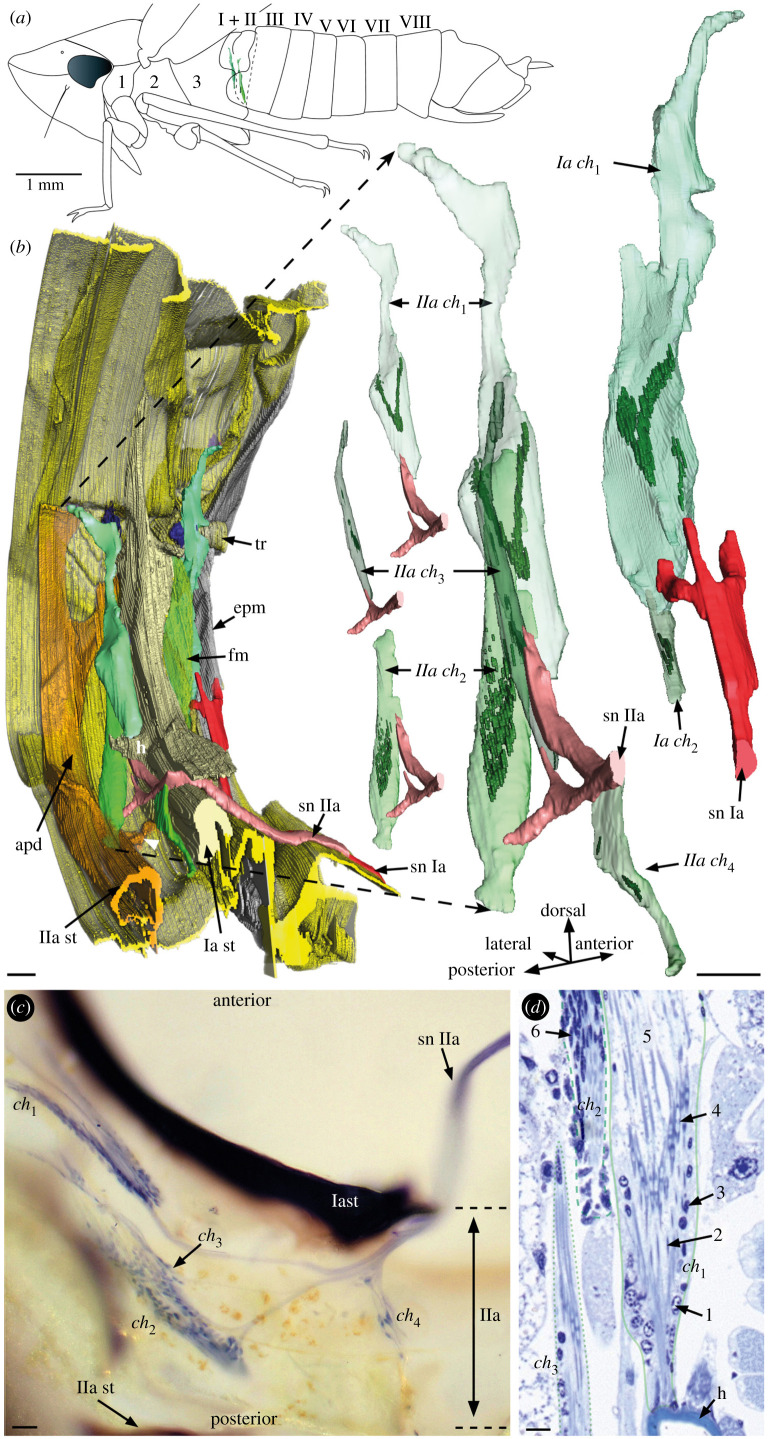
Large abdominal chordotonal organs in a *Graphocephala fennahi* male (*a*) Habitus. The first and second abdominal segments (I + II), unlike the following pregenital segments (III–VIII), are fused and contain large chordotonal organs (green). (*b*) Posterior, slightly oblique view of left hemisegment with the chordotonal organs (green), nerves (red) and exoskeleton (yellow). On the right, the chordotonal organs are enlarged with scolopales indicated in dark green. The small insets in the middle show the chordotonal organs of the second segment separately. (*c*) Dorsal view of left hemisegment. Chordotonal organs and nerves are stained with nickel chloride. (*d*) Image detail of a histological section from the second abdominal segment used for the three-dimensional reconstruction. Chordotonal organs are cut longitudinally. Units of the scolopidia are numbered: 1: soma; 2: elongated dendrite with ciliary root; 3: nucleus scolopale cell; 4: scolopale; 5: attachment cells; 6: nucleus attachment cell. Abbreviations: Ia/IIa: first/second abdominal segment; apd: apodeme; *ch*, chordotonal organ; epm: metepimeron; fm: folded pleural membrane; h: horn-like protrusion; sn: segmental nerve; st: sternite tr: trachea; white arrow head: lateral ridge. Scale bars (*b*) 50 µm, (*c*) 20 µm, (*d*) 10 µm.

To generate the three-dimensional reconstruction, a male Rhododendron leafhopper *Graphocelphala fennahi* was fixated in FAE solution (15 parts 96% non-denatured ethanol, 30 parts distilled water, 6 parts 40% formaldehyde and 2 parts 50% acetic acid) and further embedded in Araldite^®^ 502 resin. The sample was cut in 1 µm thick sections using a Leica ultramicrotome and a DIATOME Histo Jumbo 6.0 mm diamond knife. Sections were stained with Richardson's methylene blue/azur II rapid stain [16]. Images were taken by means of a 3DHISTECH PANNORAMIC SCAN II slide scanner in the Institute of Pathology Charité in Berlin-Mitte, Germany. Based on 450 serial sections (figure 1*d*) and with the software Amira [17,18], we created a three-dimensional reconstruction of the anterior abdomen (electronic supplementary material, Video S1). In contrast with the µCT images taken first, the histology allowed an unambiguous identification of cells and tissues, especially sensory structures (scolopidia, nerves) and their cuticular attachments.

Additionally, we used anterograde staining with nickel salts to study nerves and sensory neurons in both sexes of *G. fennahi* (Cicadomorpha: Membracoidea: Cicadellidae), the spittlebug *Philaenus spumarius* (Cicadomorpha: Cercopoidea: Aphrophoridae) and the planthopper *Issus coleoptratus* (Fulgoromorpha: Fulgoroidea: Issidae) (figure 1*c*, electronic supplementary material, figures S1b and S2). The insects were anaesthetized by cooling on ice, decapitated, pinned ventral side up in a dish and covered with ice-cold insect saline. The metathoracic ganglion mass (consisting of fused thoracic and abdominal neuromeres) was exposed, its posterior half isolated in Vaseline, opened up by a transverse cut approximately between meso- and metathoracic neuromeres and stained with 0.5% nickel chloride in distilled water overnight at 6°C. Nickel staining was developed using rubeanic acid (1 drop of a saturated alcoholic solution added to 1 ml saline) [19]. After development, nerves and sense organs were exposed by further dissection, fixed in 4% formaldehyde, dehydrated in an ascending alcohol series and cleared in methyl salicylate.

## 3. Results

Our investigation revealed that the first two abdominal segments in *G. fennahi*, in addition to a complex array of muscles and specialized sclerites, are equipped with a large array of chordotonal organs (figure 1; electronic supplementary material, Video S1). In the first abdominal segment, two chordotonal organs were found in each hemisegment. The first chordotonal organ (*Ia ch_1_*) is the largest one and approximately 550 µm long. One-half is made of a thin distal attachment strand and the other half of a thicker proximal part. The distal part is made of connective tissue and attached to the anterior base of a convex area of the first tergum. The distal part of the organ is in touch with a big tracheal trunk whose spiracle is located directly behind the metepimeron (figure 1*b*). The proximal portion of the chordotonal organ contains the scolopidia. The attachment takes place at a junction of the metepimeron to the first abdominal sternum. Posteriorly, the large folded pleural membrane of the first abdominal segment envelops the chordotonal organ (figure 1*b*). The scolopales of the scolopidia have been reconstructed separately to visualize their arrangement within all organs. In total, *Ia ch_1_* comprises approximately 50 scolopidia that occur in two clusters: one cluster with approximately 40 scolopidia showing a V-shaped arrangement and another with approximately ten scolopidia in a dorsoventrally oriented row. The second chordotonal organ (*Ia ch_2_*) is approximately 130 µm long and made of approximately 12 scolopidia, which are arranged obliquely to each other.

The second abdominal segment contains four chordotonal organs in each hemisegment. The first chordotonal organ (*IIa ch_1_*) is about 570 µm long. This chordotonal organ contains some 50 scolopidia, which occur in a V-shaped arrangement resembling the big cluster of *Ia ch_1_* (figure 1*b–d*). It is distally attached to the tergum II and proximally to a sclerotized hollow horn that arises laterally at the posterior side of the first abdominal sternite (figure 1*b*,*d*; electronic supplementary material, Video S1). The second chordotonal organ (*IIa ch_2_*) is about 400 µm long and contains some 70 scolopidia. They also exhibit a V-shaped arrangement, albeit less clearly than *Ia ch_1_* and *IIa ch_1_*. The distal attachment point is located ventrolaterally at the second abdominal pleural membrane, right behind the first abdominal sternite. Proximally, the organ is attached to the junction of a ventrolateral ridge with the second sternite (figure 1*b*). The second sternite forms a massive hollow bulge inwards and, at the level of the lateral ridges, forms two large spoon-like and posteriorly directed apodemes that reach into the third segment. The third chordotonal organ (*IIa ch_3_*) is about 370 µm long and comprises around 15 scolopidia in a dorsoventrally aligned row. This organ is attached to the second pleural membrane and to the posterior side of the first sternite. The fourth chordotonal organ (*IIa ch_4_*) is located ventrally and contains five scolopidia (figure 1*b*,*c*). The orientation differs from the latter chordotonal organs. The distal attachment point is in the centre of the sternum, which is concave in this area. The proximal attachment is on the posterior side of the first sternum.

Staining of sensory neurons revealed homologous arrays of chordotonal organs in *P. spumarius* and even in the more distantly related *I. coleoptratus* (electronic supplementary material, figure S1 and S2). Like *G. fennahi,* the other species examined possess two paired chordotonal organs in the first abdominal segment and four in the second. With respect to position and relative sizes, the chordotonal organs are similar in all species studied. Thus, the chordotonal organs identified as *Ia ch_1_, IIa ch_1_* and *IIa ch_2_* are the larger ones and show a V-shaped arrangement of scolopidia. In all species, *IIa ch_2_* comprise the highest number of scolopidia, ranging from 70 to 100. The fourth chordotonal organ in the second segment (*IIa ch_4_*) is located most ventrally in all species examined. Only the position of organ three (*IIa ch_3_*) seems to vary somewhat. In *G. fennahi,* it is located more ventrally and in the other two species more dorsally (electronic supplementary material, figure S1b).

## 4. Discussion

Especially in the male leafhoppers, the exoskeleton and the muscles in the first and second abdominal segments are highly modified and together form the so-called tymbal organ, which generates species-specific vibrational signals [20]. Besides these sophisticated muscle configurations in the anterior abdominal segments (electronic supplementary material, Video S1), the leafhopper *G. fennahi* shows an array of chordotonal organs that, in relation to the size of the insect, is extremely large and complex. Six pairs of different chordotonal organs occur in close proximity in the first two abdominal segments. The exceptional size of the organs is also illustrated by a comparison with the femoral chordotonal organ. In many insects, it is one of the largest leg proprioceptors with up to several hundreds of sensory cells [21]. In *Graphocephala*, the femoral chordotonal organ of the hind leg, revealed as a by-product of our nerve-staining, has only some 15 scolopidia (PB 2018, unpublished results). A similar complex of abdominal chordotonal organs was previously described in the Cicadidae [22,23] and also in the Tettigarctidae [22], a relict family with only two extant species.

We hypothesize that the remarkably large abdominal chordotonal organs represent an elaborate system for receiving and discriminating communication signals from conspecifics. Substrate-borne vibrational signals travel through the plant surface from sender to receiver. Vibrational signals are generated by tymbal buckling and oscillatory up and down movements of the abdomen [24], notably without touching the substrate. Thus, the vibratory apparatus is coupled to the substrate via thorax and legs. Likewise, substrate-borne vibrations may travel the other way round to reach the sense organs in the first abdominal segments. We assume that the membranous ventral parts of the first and second abdominal segments (figure 1*b*), depending on muscle activity, are under tension and vibrate in response to surface vibrations and/or near-field waves. Abdominal sternites one and two, which are connected to the membranes, transmit these vibrations to the attached chordotonal organs.

The regular arrangement of the scolopidia in the larger organs shows morphological similarities to the tympanal organs of crickets and katydids [25]. In addition, *IIa ch_2_*, with its high number of small scolopidia, shows similarities to the proximal scoloparium of the locust femoral chordotonal organ, a known vibration receptor [26]. Considering that the generation of tymbal signals requires a sophisticated interplay of muscle activity and abdominal stiffness [24], a proprioceptive role in the signal generation for one or more of the chordotonal organs described here cannot be ruled out. However, in view of the considerable morphological differences of muscles and sclerites between the species studied here [20], one would also expect morphological differences of the associated proprioceptors. The similarities of the chordotonal organs of the different taxa argue against this assumption (electronic supplementary material, figure S1b). Likewise, the chordotonal organs do not differ between male and female cicadellids such as *G. fennahi*, although the morphology of the tymbal apparatus shows a pronounced sexual dimorphism [20]. Even the small size of *Ia ch_2_*, *IIa ch_3_* and *IIa ch_4_* does not preclude their possible role in the perception of species-specific vibrational signals: female *Drosophila melanogaster* perceive substrate-borne mating signals with a femoral chordotonal organ with 10 and a tibial chordotonal organ with only three scolopidia [27].

These results provide evidence that all taxa examined not only share a tymbal as sound- and vibration-producing apparatus [4], but most likely also homologous signal-receiving chordotonal organs. Whereas the chordotonal organs in the first segment of cicadas correspond to a major extent with the basic pattern of the other four taxa, the tympanal organ in the second segment of this taxon shows a clear specialization for the perception of airborne sound. Nevertheless, this organ also complies with the homology criteria of topology (second abdominal segment), structural complexity (associated with lateral ridges) and continuity (with the configuration in *Tettigarcta* as intermediate form). It is conceivable that abdominal sense organs perceiving vibrational signals are ancestral in Tymbalia, and that the tympanal organs of cicadas (perceiving airborne signals) represent an autapomorphy, derived by an organ homologous to the second chordotonal organ of the second abdominal segment (*IIa ch_2_*), as described here. We hypothesize that the other abdominal chordotonal organs of the cicadas also perceive vibrational signals. Such substrate-borne vibrations are produced as a by-product of their airborne signals [28]. However, a specific generation of vibrational signals produced by cicadas cannot be excluded.

Most insects investigated so far have small chordotonal organs in the abdominal segments containing only 1–7 scolopidia. In each hemisegment, there is usually one organ in the sternal and a second one in the pleural region [21,29]. So far, the only notable exceptions are the Acrididae, in which the pleural chordotonal organ in the first abdominal segment was strongly modified and became the tympanal organ with about 60–80 scolopidia [30,31] and the Cicadidae (discussed above). The complex ‘vibroscape’ [10] of the natural habitat is particularly challenging for insects that communicate via vibrational signals. Small plant-dwelling insects require the ability to discriminate between mechanical waves of different origins (e.g. weather, predators and conspecifics) and directions. Perhaps well-known vibration receptors present in all insects such as the subgenual organs in the legs were insufficient for this task and for this reason, more elaborate mechanoreceptive sense organs evolved in the Tymbalia.

Our results provide a solid anatomical basis for future physiological investigations necessary to evaluate our hypothesis that the large mechanosensory arrays play an important role in intraspecific communication. This would be a valuable addition to the growing field of Biotremology: it is expected that by combining insights from fundamental and applied research on vibrational communication, sustainable solutions for vector control in agriculture can be developed [10]. Furthermore, the results of our work pave the way for future integrative investigations on the nature of complex communication systems and their evolution.

## Data Availability

The data are published in the digital repository of the Zuse Institute Berlin (ZIB): https://doi.org/10.12752/8326, where both the data and their description can be found.
